# Possible Futures for Network Psychometrics

**DOI:** 10.1007/s11336-022-09851-z

**Published:** 2022-03-25

**Authors:** Denny Borsboom

**Affiliations:** Department of Psychology, University of Amsterdam, Nieuwe Achtergracht 129-B, 1018 WT Amsterdam, The Netherlands

**Keywords:** Network analysis, Complex systems, Exploratory data analysis, Time series, Causal models

## Abstract

This commentary reflects on the articles included in the *Psychometrika* Special Issue on Network Psychometrics in Action. The contributions to the special issue are related to several possible future paths for research in this area. These include the development of models to analyze and represent interventions, improvement in exploratory and inferential techniques in network psychometrics, the articulation of psychometric theories in addition to psychometric models, and extensions of network modeling to novel data sources. Finally, network psychometrics is part of a larger movement in psychology that revolves around the analysis of human beings as complex systems, and it is timely that psychometricians start extending their rich modeling tradition to improve and extend the analysis of systems in psychology.

The current Special Issue of *Psychometrika* on Network Psychometrics in Action has brought together novel developments in several different strands of network psychometrics: advances in models for standard psychometric data (Lee et al., 2022; Epskamp et al., 2022; Marsman et al., 2022; Brusco et al., 2022), the analysis of time series, including the effect of interventions (Ryan & Hamaker, 2022; Henry et al., 2022; Bodner et al., 2022), and the extension of network models to novel data environments (Golino et al., 2022). It is a privilege to have been offered the possibility to reflect on this impressive collection, and I thank the Editors for this opportunity.

It does not take much reflection to see that the papers collected in this special issue license a number of immediate and optimistic conclusions. First, that we have only started to develop psychometric approaches to assessing, modeling, predicting, and controlling complex psychological systems. Many of the papers in this issue raise more questions than they answer, and one can easily imagine that new literatures will build up around topics like intervention modeling (Ryan & Hamaker, 2022; Henry et al., 2022), network mixture analysis (Lee et al., 2022), and network analysis of psychometric signals taken from social media data (Golino et al., 2022). Second, that psychometricians have an important role to play in this process, as they are naturally situated at the crossroads of psychology, mathematics, and computational sciences (Wijsen et al., 2019; Wijsen & Borsboom, 2021), three disciplines that are essential for advancing network approaches. The strength of the current articles is testament to this potential. Third, that psychometrics, as a discipline, is alive and kicking: many of the authors who are now advancing network psychometrics and related approaches are early career researchers who have their careers ahead of them. This Special Issue therefore also sketches the contours of possible futures of psychometrics as a whole, and network psychometrics in particular.

In the current commentary, I will lay out a number of areas that I believe are particularly interesting and important for carving out the future paths of network psychometrics. These include the development of intervention models, advancing explorative and inferential approaches, articulating psychometric theories to underpin network analysis, and extending the network paradigm beyond the classical data types in psychometrics. In addition, I will reflect on issues that I see as problematic, such as the inadequate delineation of explorative and inferential aspects of network psychometrics, and the poor state of network theories that should serve to anchor statistical modeling approaches.

## Understanding, Prediction, and Control of Psychological Systems

In my view, the central objectives of science are understanding, prediction, and control. These are strongly related, because a better understanding of systems allows us to predict their behavior under different scenarios, and if these include interventions (as per causal inference), this harbors the possibility of control. For instance, if we understand the network structure and dynamics of a disorder like depression, we can work out how different interventions change the system (Lunansky et al., 2021; Henry et al., 2022; Ryan & Hamaker, 2022), which would allow the selection of optimal interventions that can be used to alleviate a person’s problems (Rubel et al., 2018).

So far, network approaches have mainly contributed to a better understanding of psychological phenomena, in the sense that the conceptualization of psychological constructs as networks has offered a new frame of thinking about them that, in many cases, seems to carve nature at least slightly closer to its joints. These benefits have been most clearly surfaced in the study of intelligence (van der Maas et al., 2006; Savi et al., 2019), attitudes (Dalege et al., 2016), psychopathology (Cramer et al., 2010; Borsboom & Cramer, 2013), and personality (Costantini & Perugini, 2012). In addition to this conceptual advance, network analysis offers an explorative approach to mapping out the structure of the proposed networks on the basis of data (Borsboom et al., 2021), and this application of network ideas is arguably the most important reason for its current popularity (Robinaugh et al., 2020).

However, conceptual work and explorative data analysis can only take us so far. For instance, if we want to develop network models that can help in guiding, monitoring, and evaluating treatment of mental disorders, we will have to develop ways of incorporating controlled interventions into the analysis. Examples of work that has taken the first steps in this direction include Blanken et al.’s (2019) network intervention analysis, Waldorp et al.’s (2021) perturbation analysis, and Lunansky et al.’s (2022) simulation based approach. Neither of these, however, are suitable to continuous monitoring and evaluation of interventions based on time series, which arguably is a primary candidate for enriching therapeutic practice. The work of Henry et al. (2022) and Ryan and Hamaker (2022) marks a considerable step forward toward methodologies that may ultimately evolve to guide intervention processes in this way.

Henry et al. (2022) present an application of control theory to integrated vector autoregressive networks. Although, as the authors correctly remark, these analyses are still in their infancy and will require important extensions to deal with the different time scales and nonlinearities that we may expect in real situations, I consider their paper to be a landmark achievement. Despite the limitations of the models used, Henry et al. (2022) present a systematic approach to the application of control theory that I expect will spark an important research program that can serve to extend psychotherapists’ cockpit by improving their vision, planning, and monitoring possibilities.

Ryan and Hamaker (2022) take a similar track, but in addition address the important issue that effects should be evaluated as they play out in continuous time. Continuous time models will become important in the future, because interactions between variables in, e.g., psychopathology applications, are likely to work at different time scales; one only needs consider the fact that emotional responses play out at the level of minutes or even seconds, while sleep processes work at the day-to-day time scale, and crucial learning processes can take months or even years. In addition, in contrast to standard vector autoregressive approaches, which presume that time intervals between measurements are equal, Ryan and Hamaker’s (2022) model can naturally address situations where no fixed schedules are used to gather data; an example, which I expect will become prevalent in future research, concerns observational data based on spontaneous actions (e.g., observations of relevant behavior, like leaving the house, or posting Twitter feeds; Golino et al., 2022).

Developing, testing, and improving applications of this type of work to therapy would seem to be of immediate relevance to the areas of clinical psychology and psychiatry. The development of intervention models may also serve to structure discussions on which features of networks identify viable targets for intervention (Rodebaugh et al., 2018). In particular, the centrality measures of (Ryan & Hamaker, 2022) should be helpful in assisting clinical researchers in this respect. In addition, extensions of the approaches of Ryan and Hamaker (2022) and Henry et al. (2022) to related intervention situations such as persuasion (Zwicker et al., 2020) and education (Savi et al., 2019) would seem to be fruitful avenues to explore. Together with other recent approaches (Robinaugh et al., 2020; Lunansky et al., 2022; Blanken et al., 2019; Waldorp et al., 2021), a considerable set of methodologies to approach the analysis of interventions is now building up. I expect that the methodological study of these approaches as well as their substantive application will define an important research area in network psychometrics in the next decade.

## Exploration and Inference in Network Psychometrics

In my view, network psychometrics originates in the tradition of exploratory approaches, in the sense that its origins are closer in spirit to exploratory data analysis (Tukey, 1977) than to traditional methods of statistical inference (Neyman & Pearson, 1967). After all, the original aim of using network representations of psychological constructs was not so much to estimate a model, but to depict statistical associations visually, in order to help researchers generate hypotheses on network structures that could be useful in further research (Cramer et al. 2010; Epskamp et al., 2012). The visualization of network structures is still an essential part of the network psychometric paradigm, as it offers a mode of representing modeling results that is unique in its power to engage with substantive users of the techniques. However, as has been documented, aesthetically pleasing representations such as the Fruchterman–Reingold algorithm (Fruchterman & Reingold, 1991) need not always be optimal in representing statistical associations and network structures built on them (Jones et al., 2018). As such, the matrix permutation method of arranging networks developed by Brusco et al. (2022) offers an important network representation to maximize information transfer to the user of these techniques.

The application of regularization, as developed in van Borkulo et al. (2014) on the basis of techniques suggested in Foygel and Drton (2010), brought methods of network analysis into the realm of model selection, which led to generic model formulations that may serve to underpin network psychometrics (Epskamp et al., 2017). Regularized estimation, as now commonly used in network analysis (Robinaugh et al., 2020), minimizes a penalized function of the likelihood (e.g., the extended Bayesian Information Criterion) and as such operates by following a tractable search path through the model space on the basis of neighborhood selection, either through nodewise regression approaches (van Borkulo et al., 2014; Haslbeck & Waldorp, 2020), by penalizing the partial correlation matrix in one single sweep (Epskamp & Fried, 2018), by adaptively tuning the penalization to data characteristics and inferential goal (Wysocki & Rhemtulla, 2021), or by using a combination with stepwise selection (Isvoranu et al., in press). The advantage of such approaches is that it is clear what mode of exploration is followed. That is, it does not encourage tinkering under the hood, as is for instance common in Structural Equation Modeling and other traditions, where researchers sometimes follow an opaque search path through the model space, typically by changing model parameters in a haphazard way until they find model fit acceptable. Regularization approaches structure this process and can identify an optimal model for the data in terms of the specific balance between fit and parsimony that is defined explicitly in the methodological setup. Of course, one can criticize the criteria used, but the value of transparency in the explorative research process should not be underestimated.

In this perspective, regularized network analysis defines an explorative search for a restricted model that fits the data best in a clearly defined sense. However, as Marsman and Rhemtulla (2022) suggest, regularized approaches can also be interpreted differently, for instance, as procedures aimed at estimation or inference, and with respect to these goals they can have considerable downsides. For example, regularized estimates are often biased and their sampling distribution is unwieldy. In addition, in certain cases, regularized estimation through the graphical lasso can go awry (Williams & Rast, 2020; Wysocki & Rhemtulla, 2021) although I personally find the contrast between results from different simulation procedures hard to make sense of and doubt the generality of some of the claims now in the literature (e.g., compare the results of Williams & Rast, 2020, to those of Isvoranu & Epskamp, 2021). Finally, if the generating model is not sparse but dense, the regularized approach can struggle to approximate the generating model (Epskamp et al., 2017), which is especially problematic if edges that have been suppressed in the estimation process are used inferentially, namely as evidence for the absence of links in the generating network—the well-known credo that absence of evidence is not evidence of absence applies here as it does elsewhere (Marsman & Rhemtulla, 2022; Marsman et al., 2022).

In view of these limitations, after the initial dominance of the regularization approach, we now see a number of alternative ways to determine, estimate, and represent network structures in psychometric applications. One alternative is to construct networks on the basis of different statistical information, as is done in information filtering (Christensen et al., 2018), and in the Jaccard index approach of Bodner et al. (2022). These procedures build networks on qualitatively different principles, as they do not utilize conditional associations as building blocks. Another innovation that goes beyond explorative search algorithms is provided by the methodology of Marsman et al. (2022), which can help to evaluate evidence of absence, and as such adds to a burgeoning literature that develops methods to assess the confidence we should place in the presence and absence of edges in an estimated network which, given common interpretative practices, are important determinants of network structure. Finally, the meta-analytic framework of Epskamp et al. (2022) allows for the aggregation of evidence across studies, which is crucial in assessing robustness and generalizability of networks. Taken together, these approaches both widen the options available for estimation and inference, and offer additional tools to improve the robustness and generalizability of network analysis.

However, as they say, with one watch one always knows the time but with two watches one never is quite certain. The proliferation of alternative network analysis and representation techniques thus yields an important new problem, namely what method to choose from this pantheon. I would be hesitant to buy into a general dogmatic position here, and would favor the idea that the choice of model should be dependent on the goals of the researcher and the substantive context. In particular, I would suggest that regularized estimation is useful for explorative model search, nonregularized estimation with significance testing is useful to control Type I errors, and Bayesian methodology is useful to evaluate the evidence for edge exclusion.

However, it will likely be unclear to many researchers exactly how goals and context should be coordinated with choice of analysis. There is, in my view, therefore a need for papers that assist researchers in making this choice. Tools such as those provided by Isvoranu and Epskamp (2021), who proposed an interactive app to make it easy for researchers to evaluate the pros and cons of different methods, are highly useful in this respect, as are adaptive techniques to tuning as developed in Wysocki and Rhemtulla (2021). Also, the prospect of combining different models, analogous to how this is done in model averaging (Hinne et al., 2020), is attractive from the perspective of robustness.

## The Inferential Target

By moving from simple depictions of correlation structures to the search for an optimal model, network approaches have taken on some of the characteristics of inferential statistics: techniques are now commonly interpreted and evaluated for their ability to recover a generating structure or to decide between rivalling explanatory models (van Borkulo et al., 2014, 2019). They have thus migrated from exploratory to at least partly inferential, and critiques of the model typically concern this aspect (Williams & Rast, 2020).

Inferential statistics needs an inferential target: in this case, a data-generating network. Indeed, the notions of sensitivity and specificity, which are often studied to evaluate network recovery, can only be meaningfully applied under the prior assumption that there actually is such a generating network. There is a significant tension here for every model, but this is especially pronounced for regularized approaches, because it is evident that the inferential assumptions necessary to guarantee the convergence of such approaches on the truth will rarely be met in realistic psychometric scenarios; in particular, it is unlikely that psychometric networks are sparse (Hallquist et al., 2021; Epskamp et al., 2017; Williams et al., 2021). Thus, notwithstanding the explorative uses of standard regularized approaches, when understood as inference techniques, they can run into problems.

On the other hand, there are good reasons to be skeptical about whether inference should be a direct goal for network approaches in most applications. The putative existence of a “true generating model,” for instance, seems to be an ambitious assumption. While networks probably do a better job of representing the complexity of psychological constructs than traditional psychometric approaches, in many applications they are still likely to be gross oversimplifications. Will any partial correlation ever be zero in the population (whatever it may be)? Do we ever encounter a situation in which random sampling, assumed in the background of most inference techniques, has been realized? Are we serious in presuming homogeneity, when it is likely that the networks we estimate reflect partly heterogeneous populations that may be better characterized as mixtures (Lee et al., 2022)? Can we assume that the statistical properties of network estimates are covered adequately, when almost all networks are likely to feature missing nodes? And what about various idealizations that are necessary for the statistical modeling framework, but that will preclude even the correct specification of the generating model in the first place (e.g., distributional assumptions, linearity, the strict absence of higher order interactions)? The challenge may thus not be to arrive at the correct model, but at the model that is most conducive to improve our understanding of the systems studied.

What these questions bear out is that scientific inference is not the same as statistical inference. Statistical inference only considers the adequacy of a method in idealized conditions, and presumes the many additional scientific issues to have been fixed. Scientific inference, in contrast, must consider many relevant issues outside of the statistical framework (Borsboom & Haig, 2013).

Statistical models often proceed from a thought experiment in which mathematically tractable structures generate idealized observations that are studied in pure random samples. This is useful, because it allows us to study the methodology for its strengths and weaknesses; arguably, there is no other way to investigate the systematic long run behavior of our modeling procedures. However, thought experiments are not real ones. Playing the thought experiment “what if this model generated the data and we applied technique x to these data?” can be conducive to the evaluation of the inferential adequacy of a methodological technique. Nevertheless, we should be careful in transferring inferential results from our idealized mathematical realm to the messy realities where we apply them. In setting up statistical techniques, we use toy inferential targets to evaluate the performance of our methods. While these hopefully capture central aspects of the systems studied, they are not the real thing. So what, then, *are* our scientific inferential targets? And how do statistical models relate to them? To answer this question, we only have one possibility at our disposal: we have to build scientific theories of psychometric constructs.

## Theories and Models

The above reflections on the difference between plausible architectures of actual psychological systems and the structure of common statistical data models feed into to another issue that is highly relevant in network psychometrics, namely the limited attention to psychometric *theory* as compared to psychometric *analysis*. I have noted the problems of disconnect between substantive theories and psychometric methods that can arise from an exclusive emphasis on data analysis in earlier work, and have instigated psychometricians to get involved in theory formation (Borsboom, 2006); the development of the network theory of psychopathology (Borsboom, 2017) was in fact an attempt to put my substantive money where my psychometric mouth was. However, even though the development of theory on the organization and dynamics of the systems under study has been much more forthcoming than I originally anticipated, it is dwarfed by the tremendous speed and force with which new data analysis methods have been developed and applied (Robinaugh et al., 2020).

As a result, network analysis is at risk of outpacing network theory in much the same way that latent variable modeling outpaced latent variable theory in the previous century. In my view, an important problem that contributes to this process is that our discipline tends to confuse statistical and scientific inference, i.e., it has a habit of mistaking elements in the statistical model for targets of the substantive theory. This easily leads to a situation where the inferential target, as noted in the previous section, is simply proposed to be a specific parameterization of the statistical model, which then acts as data generating mechanism in proofs and simulations. As a result, the question of inference is reduced to the question of how well this model could be recovered from data in idealized circumstances. I do not contest the usefulness of that information, but do suggest it would be highly useful if more attention was devoted to actual development of the substantive theoretical model, as this can bring out differences with our statistical model that remain invisible if we view the situation exclusively from within the statistical realm.

For example, take the case of depression. As soon as we start thinking about what actually is the scientific rather than the statistical target of inference in depression models, we see that these will rarely be exactly the same. In an early and important contribution to network approaches, Wittenborn et al. (2016) constructed an elaborate theoretical structure based on the literature on depression. This structure contains a mixture of psychological processes (e.g., rumination, feelings of guilt), biological features (e.g., neurotransmitter levels, structural changes in certain brain regions), and social factors (e.g., interpersonal relationship quality, financial stress) that play out on different levels and at different time scales. In contrast, network analysis models have invariably been fitted to simple symptom sets or items from designated depression questionnaires, in which symptoms are typically variables presumed to have the same scale, and in which all links between symptoms are conditional association parameters. I have no idea whether Wittenborn et al.’s (2016) theory is correct; this is not important for the current discussion. The point is that no statistical model fitted to a set of symptom values could possibly recover this type of inferential target.

Similarly, Robinaugh et al. (2019) include various features into their model of panic disorder (e.g., anxiety sensitivity, escape behavior, different time scales at which feedback loops interact) that are never assessed (even indirectly) in the standard symptom data fed into statistical network models for panic disorder. How could the data-analytic model correctly retrieve a model that is not even a possible outcome of an estimation algorithm? Clearly, in such cases, the relation between data models and theoretical models is indirect. In cases where it is possible to construct a correspondence between theory and model, that should of course be a first choice, as it aligns statistical with scientific inference (Rhemtulla et al., 2020). However, in cases where no such alignment is forthcoming, we need more than statistical inference techniques to make the connection between theory and data (Haslbeck et al., 2021). To presume that inference, in such cases, is just a matter of selecting the right statistical model is an oversimplification that can obstruct progress.

It is sometimes suggested that dynamical models of time series overcome some of these issues, as they have a more direct relation to the processes of interest. But this is an idle hope. Staying within the context of psychopathology, exactly the same sort of mismatch occurs in time series analysis, which is usually executed at the level of momentary mood states. Even though researchers sometimes label these as symptoms, they cannot be mapped directly to the symptomatology of disorders as a matter of principle. For instance, time series analyses typically proceed by filtering out person-specific means first and analyzing only the deviations from these means. However, symptoms are assessed in terms of mean levels, as even superficial perusal of the DSM-5 shows. Similarly, if there are trends in the time series, these are typically subtracted from the observations to meet the requirement of stationarity. However, in several cases (e.g., psychopathology; Borsboom, 2017), network *theory* is about precisely the relations between the trends that are filtered out of the network *model *(e.g., how increasing sleep loss ultimately produces increasing levels of fatigue). To illustrate the issue in examples taken from this Special Issue, hypotheses that pertain to symptom evolution as modeled in Bodner et al. (2022) need not carry over to time series models for momentary mood states, as modeled in Henry et al. Fried (2022).

As in the examples of depression and panic disorder above, the lack of a clear mapping between the components of the theoretical and statistical models is a real problem here. This problem should be addressed if we are to use the modeling innovations of the past years to actually advance our understanding of the systems under investigation. The construction of psychometric theories (i.e., substantively informed formal models) in addition to psychometric models (i.e., content-neutral statistical modeling tools) is therefore an essential prerequisite for both advancing our substantive understanding of psychological systems and developing a better view of how network modeling techniques behave under different theoretical scenarios. To assess statistical inference in modeling techniques, we study how they would behave if a given model were true; to assess how they work in the case of scientific inference, however, we need to understand what our models do if our theories are true (Haslbeck et al., 2021). Bayesian techniques, such as those in Marsman et al. (2022), could then perhaps be used to motivate prior distributions for data models based on expectations generated by theoretically informed generative models (Haslbeck et al., 2021).

Why do I go through such lengths to make this point? Because I see a possible future in which diverse strands of network psychometricians battle each other over whether we should be Bayesian or frequentist, favor estimation or testing, analyze time series or cross-sectional data, fit the model to the data or fit the data to the model, be subjectivist or objectivist, use regularized or non-regularized estimation, etc. If we are considering possible futures for our emerging discipline, I would like to take the opportunity to issue a vote against that one. Different methods and techniques answer different questions and give different types of information. In my view, the primary objective should not be to decide which method is uniformly best, but learn how we can utilize of these different kinds of information to advance our understanding.

## Networks in the Era of Social and Behavioral Data Science

Network psychometrics arises out of a non-traditional way of approaching traditional data, i.e., data that concern responses of subjects to questionnaire items or interview questions. Examples include the dichotomous presence of symptoms, ordinal responses to Likert scales, and continuous assessments using visual analogue scales, as recorded across or within subjects (Borsboom et al., 2021).

However, the broader notion of conceptualizing constructs as networks invites the use of other modes of observation, which generate other kinds of data and require other kinds of models. An early extension of this type concerns the analysis of time series of momentary mood states (e.g., experience sampling and other forms of mobile assessment; (Bringmann et al., 2013), but other examples include results from activity monitoring (e.g., actigraphy data, location data), posts on social media (Kelley & Gillan, 2022; Golino et al., 2022), intervention data (Blanken et al., 2019; Waldorp et al., 2021), data on interactions with other individuals (Bodner et al., 2021), and data gathered at different levels of observation (e.g., registrations of brain activity, genetic data, social network data; Blanken et al., 2021).

Fortunately, the Special Issue includes important extensions of network models that foray into these new worlds of data. Bodner et al. (2022) contribute new approaches to the analysis of categorical time series, and Golino et al. (2022) extend exploratory graph analysis methods to analyze social media data. Golino et al.’s (2022) approach yields a highly interesting new way of using network analysis on qualitative bodies of data that I imagine could potentially have many applications, such as attempts to assess network structure based on the analysis of features of intra-individual time series of Twitter posts (Kelley & Gillan, 2022), and could also serve to bridge the traditionally deep divide between qualitative and quantitative data.

This broadening of our observational horizon is not only a welcome development for network approaches, but also for psychometrics itself. In my view, psychometrics has limited its domain of study to the analysis of traditional data (pencil-and-paper questionnaires) and simple extensions thereof (computerized testing) for too long. It is high time that psychometricians start engaging with the tsunami of data that is approaching us; for twenty-first century social science data will not fit the twentieth century psychometric toolbox. I anticipate that before long, analysis of these new kinds of data will start to play an important if not dominant role in the psychological literature, and it is urgent that psychometrics extends its scope to deal with them. Traditional methodologies, which typically involve storing a static dataset on a computer and analyzing it with standard statistics, are ill suited to deal with this new situation, in which huge, continuously updated databases rather than static data files will be the norm. It will therefore be essential to utilize methodologies that are now being developed in data science (Buyalskaya et al., 2021).

Network analysis is naturally suited to connect different kinds of data and levels of analysis (Blanken et al., 2021; van der Maas et al., 2020) and as such may play a prominent role in the emerging era of social and behavioral data science. However, to take full advantage of the increasing availability of data will require new extensions the network paradigm. For instance, if network psychometrics is to engage with complexity science at large, we will have to work toward methodologies that are suited to connect network psychometrics to, for instance, network neuroscience and social network analysis (Blanken et al., 2021). In addition, the trove of possibilities that network science affords generates many additional possibilities for analyzing network structure which have hardly been touched; Brusco et al.’s (2022) application of matrix permutation methods is a case in point, but there are many other possibilities, including the use of multigraphs (Brooks et al., 2020), minimal spanning trees (Letina et al., 2019), integrated network models (Blanken et al., 2021), and network-based regression (Bathelt et al., 2021).

Integrating information from different and quickly expanding data sources thus is an important theme for the future of network psychometrics. Indeed, several of the papers in the Special Issue touch upon it. Espkamp et al., (2022), for instance, extend the network paradigm to meta-analytic techniques that integrate data from different studies; Bodner et al. (2022) venture novel ways of integrating time series data from different participants (see also Gates & Molenaar, 2012); the methods of Lee et al. (2022) can be used to decompose cross-sectional data into possibly generative clusters; and the approaches in Ryan and Hamaker (2022) and Henry et al. (2022) may be used to integrate experimental and observational data. In time, we also need to develop ways of informing theoretical models (Robinaugh et al., 2019; Wittenborn et al., 2016) with these and other sources of data. This will require innovations in how to connect and integrate findings from disparate sources of information into the same model. The way theoretical simulation models are used in other disciplines can provides useful templates for integration in this respect. For instance, just like the International Panel on Climate Change (IPCC) integrates many different sources of information and modeling into a larger climate model, we have to come up with ways to integrate information from several modes of observation into theoretical models that capture the structure and dynamics of psychometric constructs.

## Conclusion

I compliment the Editors for building a collection of articles that define a research agenda for years to come. As I have argued in this comment, major themes on this research agenda involve further development of time series models, intervention analysis, exploratory and inferential techniques, theory formation, and extensions of network modeling to novel data sources. In closing, I would like to offer some additional suggestions on where all this fits in the bigger picture and relate network psychometrics to the historical record.

Psychometrics has traditionally been assigned to the correlational half of psychology, as per Cronbach’s (1957) famous division of scientific psychology into two strands of research that rarely meet—correlational and experimental psychology. However, it is interesting to note that, when conjoined with intervention modeling (Henry et al., 2022; Ryan & Hamaker, 2022), network analytic approaches naturally combine patterns in correlations with shifts in means. Thus, they build bridges between the experimental tradition of zooming in on intervention effects (usually built entirely on the analysis of mean differences between conditions) and the correlational tradition of analyzing relations between variables (traditionally built on multivariate dependencies in observational data). In a nontrivial sense, in time these approaches may therefore serve the long overdue goal of connecting Cronbach’s (1957) disciplines in a systematic overarching methodology.

Another important dimension concerns the issue of parts and wholes. Throughout the history of psychology, our science has swung back and forth between reductionistic and holistic approaches to understand human beings (e.g., see Brysbaert & Rastle, 2020). The decades behind us were an age of reductionism, characterized by attempts to analyze human behavior in terms of lower levels of explanation (e.g., neuroscience, genetics), by isolating psychological processes in dedicated laboratory tasks, and by breaking down the human system in terms of constituent elements. These approaches have yielded much of our current knowledge, but they have considerable limits. Humans fundamentally are interconnected systems, and to build a science of the human being as a whole, we have to find ways of modeling our research topic at that level. Experimentally speaking, the isolation of processes and causal links is essential, but theoretically speaking, the complex systems view is the only game in town.

The complexity perspective takes psychometrics into new territory. As this Special Issue may well mark the arrival of a new psychometric tradition that forays into that territory, it is important to adequately document its roots before they fade from memory. In this respect, I would like to draw attention to an important historical element behind the development of network approaches as discussed by Marsman and Rhemtulla (2022), namely the influence of Peter Molenaar. Molenaar was the first to understand and appreciate the resemblance between network models used in statistical physics (particularly the Ising model) and psychometric models (particularly, Item Response Theory). In fact, Molenaar (2003, p. 82) already suggested that the Rasch (1960) model is equivalent to the Ising model (Ising, 1925; Niss, 2005), which was later proven to be indeed the case (Epskamp et al., 2018; Marsman et al., 2018). Arguably, this type of equivalence actually lies at the basis of the network approach, which started as an alternative way of explaining correlation structures that were typically seen as evidence for latent variables (van der Maas et al., 2006). Second, Molenaar (2004) championed idiographic $N=1$ studies using time series in a time when no living soul in psychology or psychometrics recognized the importance of such techniques, and many of the current network models for time series analysis are indebted to his pioneering work (see also Molenaar et al., 2003). Third, the idea of using control theory to guide therapy, which is now on the horizon thanks to the work of Henry et al. (2022) and Ryan and Hamaker (2022), was articulated and implemented by Molenaar as early as 1987 (Molenaar, 1987). Finally, Peter Molenaar has been a mentor to many of the leading contributors to the network approach on both sides of the Atlantic. In many ways, network psychometrics is thus indebted to his vision and ideas.

The increasing popularity of network modeling fits a larger movement in psychology. It is a movement that emphasizes connections between components that were traditionally studied in isolation, recognizes qualitative as well as quantitative differences between people, engages with the time evolution of systems, actively integrates different modes of observation and levels of analysis, and has the relation between structure and dynamics of psychological systems as a central research topic. Although network analysis is but one of a variety of techniques that are important within this emerging perspective, it is an important one and the methodologies developed in this Special Issue position it right in the center of psychometrics. I hope that the psychometric community will engage with this material, and will use psychometrics’ rich heritage to improve and extend the analysis of complex systems in psychology.
